# Source-Dependent Structuring of Hydrogen-Oxidising Bacterial Community Composition During Enrichment and Isolation from Freshwater Environments

**DOI:** 10.3390/microorganisms14061221

**Published:** 2026-05-28

**Authors:** Emine Gozde Ozbayram, Marcell Nikolausz

**Affiliations:** 1 Department of Marine and Freshwater Resources Management, Faculty of Aquatic Sciences, Istanbul University, 34134 Istanbul, Türkiye; gozde.ozbayram@istanbul.edu.tr; 2 Department of Microbial Biotechnology, Helmholtz Centre for Environmental Research–UFZ, 04318 Leipzig, Germany

**Keywords:** gas-utilising bacteria, hydrogen-oxidising bacteria, isolation, protein, selective culturing

## Abstract

This study set out to cultivate and isolate hydrogen-oxidising bacteria (HOB) for microbial protein production under a specific culture strategy with a particular focus on assessing the influence of different environmental sources on enrichment culture and strain diversity. Therefore, HOB were enriched from samples collected from various freshwater lakes and streams, and novel strains were subsequently isolated from these cultures. The enrichment procedure revealed significant shifts in community compositions, which were mainly driven by changes in the relative abundance of genera affiliated to Pseudomonadota and Bacteroidota. Sample-specific variations were observed in the communities of the inocula, reflecting distinct community structures associated with distinct ecological functions. The most common autotrophic HOB, *Hydrogenophaga*, proliferated in some of the cultures. However, several genera, such as *Acinetobacter* and *Klebsiella* that have not been previously recognised with hydrogen-oxidation characteristics, were also enriched, suggesting potential novel contributors to HOB communities.

## 1. Introduction

Microbial protein is the dried microbial biomass which can be produced by various microorganisms, including bacteria, fungi, yeast, and algae, and utilised as a food or feed [1,2,3,4]. It is typically rich in nutrients, including a substantial protein content (reaching up to 75% of its dry biomass) with essential amino acids, vitamins, and minerals [5]. In an era of rising global population, microbial protein is considered an environmentally friendly substitute for conventional plant and animal-derived proteins [3] and has gained growing scientific and commercial interest [1,6,7,8,9,10,11]. The use of microbial protein has potential positive contributions towards meeting the United Nations Sustainable Development Goals (SDGs). First, microbial protein could support food security (SDG 2), which offers a promising alternative to traditional farming, particularly in areas where affordable protein sources are scarce [12]. Further, it can potentially reduce reliance on conventional animal feed components such as soybeans [13], fishmeal [14], and various cereals [15], a major component of animal feed formulations. The use of microbial protein could mitigate the need for expansion of agricultural lands for soybean cultivation and align with the goals related to biodiversity preservation (SDG 15). The utilisation of autotrophic microorganisms for microbial protein production could contribute to the reduction of greenhouse gas emissions (SDG 13). Additionally, incorporation of microbial protein into feed formulation could ease the pressure on fish stocks, leading to more favourable conditions for marine ecosystems (SDG 14) [7]. In addition to its use in animal feed, microbial protein is considered as a potential food source for space exploration, thereby expanding the range of applications for microbial protein production [16].

Hydrogen-oxidising bacteria (HOB), also known as *Knallgas* bacteria, are aerobic chemoautotrophs that use H_2_ as the electron donor and O_2_ as the electron acceptor, and fix CO_2_ as a carbon source through the Calvin cycle or reverse Krebs cycle [3,17,18,19]. They can rapidly grow on basic cost-effective media with gaseous substrates [17]. HOB are phylogenetically very diverse with representatives belonging to various phyla, including Proteobacteria (Pseudomonadota according to the novel taxonomy), Firmicutes (Bacillota), and Actinobacteria [20]. A pioneer study carried out in [21] demonstrated this diversity by identifying HOB members spanning both Gram-negative genera such as *Alcaligenes*, *Pseudomonas*, *Paracoccus*, *Aquaspirillum* and *Flavobacterium*, and Gram-positive genera including *Corynebacterium*, *Nocardia*, *Mycobacterium*, and *Bacillus*. They also have various life history strategies, including mixotrophy, so they can also use organic carbon [22]. Analysing bacterial strains capable of oxidising molecular hydrogen in the presence of oxygen has provided extensive insights into their physiology, metabolism, and kinetic properties. A significant milestone in this field was obtaining the genome sequence of *Cupriavidus necator* [23], which is considered a model HOB [19]. The protein content in HOB (75%) is by far greater than that of algae (50%), soybean (45%), fungi (40%), and wheat grains (15%) [19].

The diversity and adaptability of HOB make them well-suited candidates for large-scale production of microbial protein [8]. Although further optimisation is still required to fully establish HOB as robust and efficient cell factories, numerous species already exhibit considerable potential for genetic modification and industrial biotechnology applications [24]. Moreover, Life Cycle Assessment studies suggest that utilising HOB for microbial protein production, coupled with low-emission sustainable energy, can significantly reduce GHG emissions and land use, while also contributing to mitigating eutrophication of surface waters compared to conventional plant or animal protein production. This approach has the potential to lower environmental impacts by more than 50% [19].

Potential environments supporting the chemolithotrophic growth of *Knallgas* bacteria include the rhizosphere of leguminous plants, surface waters in marine settings, geothermal locations, and microbial mats predominantly composed of cyanobacteria [25]. However, *Knallgas* bacteria in pure culture mostly originate from soil.

Despite the growing interest in HOB for sustainable microbial protein production, a critical gap remains in understanding how the origin of environmental inocula influences enrichment culture structures and the diversity of cultivable species. Particularly, systematic investigations linking source variability to community assembly and isolate recovery under defined cultivation conditions are lacking. Additionally, many existing studies rely on technically complex enrichment systems, highlighting a further gap in the development of simple and reproducible strategies applicable across diverse settings.

Therefore, to address these gaps, this study investigates how distinct freshwater sources shape the diversity and composition of HOB enrichment cultures and the strains subsequently isolated, using a simple and reproducible enrichment and isolation strategy.

HOB were initially enriched from nine different samples collected from various freshwater lakes and streams, and the bacterial community dynamics of the enrichment cultures were monitored using amplicon sequencing. This approach enabled the identification of source-dependent selection patterns and facilitated the isolation of previously uncharacterised novel HOB strains that were taxonomically affiliated based on partial sequencing of the 16S rRNA gene.

## 2. Materials and Methods

### 2.1. Enrichment of Hydrogen-Oxidising Bacteria

A total of ten environmental samples were taken from freshwater resources, including three streams, three lakes and one pond, comprising different sample types such as sediment, algae mat, and rhizosphere material. Samples were collected in a sterile 50 mL tube filled with 25 mL of sample material, then topped with source water (Table 1). Note that S03 and S04 represent two replicate samples collected from the same site (Yanikdere Stream) on the same date, and were included to assess the reproducibility of the enrichment process and to evaluate within-site variability under the same environmental conditions.

Enrichment was carried out in 250 mL bottles closed with butyl rubber stoppers with an active volume of 100 mL, including 10 g of sediment and 90 mL of sterile medium DSMZ81 (Table A1). The headspace of the bottles was first flushed with sterile air, then over-pressurised with H_2_/CO_2_ (83/17%) to reach 0.8 bar over atmospheric pressure (with theoretical gas composition of O_2_/N_2_/H_2_/CO_2_ = 11.7/43.9/36.8/7.6). The cultures were incubated at 28 °C and stirred continuously in an orbital shaker (200 rpm). The first enrichment and two subsequent transfers (Transfer 1 and Transfer 2) each lasted 10–15 days depending on hydrogen consumption, and were investigated. The gas pressures were monitored with a high-resolution manometer (LEO 5, Keller, Winterthur, Switzerland) and the gas compositions were measured by gas chromatography (InFicon Micro GC Fusion^®^ Gas Analyser, INFICON, Bad Ragaz, Switzerland) equipped with a thermal conductivity detector with the columns Rt-Molsieve and Rt-Q-Bond with the column temperatures 80 °C and 60 °C, respectively. During the incubation of both transfers, the headspace gas was replenished two times by flushing first the headspace with sterile air and then by over-pressurising with H_2_/CO_2_ as described above.

### 2.2. Isolation of Hydrogen-Oxidising Bacteria

**Figure A1:**
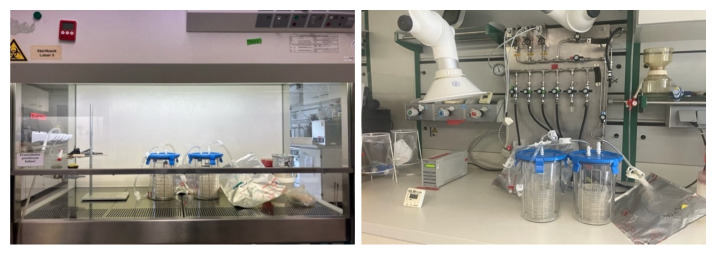
Preparation of plates for incubation in a modified anaerobic jar with an attached gas bag.

Six enrichment cultures (HE02, HE05, HE06, HE07, HE08, and HE10, corresponding to samples S02, S05, S06, S07, S08, and S10, respectively) were selected for further isolation approach based on elevated hydrogen consumption. To obtain HOB, the samples from those enrichment cultures were subjected to serial dilution (10^−1^ to 10^−8^). Mineral medium agar plates (DSM 81) were used for the isolation (Appendix A, Table A1), and 100 µL samples were plated from the dilution steps (10^−5^ to 10^−7^) using the spread plate method. The inoculated plates were then placed in custom made incubation jars (2.5 L) connected to a gas bag filled with a mixture of CO_2_:H_2_ (1:4.8, 1.8 L), which together with the air in the jar provided a proper mixture for HOB autotrophic growth (exact gas composition is influenced by the number of plates incubated in the jar). The jars were incubated at 28 °C for one week for autotrophic aerobic growth (Appendix A, Figure A1). Subsequently, 16 colonies from the highest dilution plates were picked with a sterile loop and transferred to a new agar plate under sterile conditions. Pure cultures were obtained by the streak agar technique, and pure colonies from the agar plates were also transferred into liquid media.

### 2.3. Microbial Community Analysis

#### 2.3.1. 16S rRNA Gene Amplicon Sequencing

For DNA isolation, 0.5 g samples were collected from the sediments. From the enrichment culture and subsequent transfers, 2 mL liquid samples were collected, centrifuged at 20,817× *g* for 10 min, and the supernatant was removed. All samples were stored at −20 °C. The total DNA were isolated from the sediments and pellets using NucleoSpin^®^ Soil Kit (Macherey-Nagel GmbH & Co. KG, Düren, Germany) with SL2 buffer. The concentration and quality of the isolated DNA were determined using a NanoDrop spectrophotometer (Thermo Fisher Scientific, Waltham, MA, USA) and 0.8% agarose gel electrophoresis.

The bacterial communities of the inoculum (sediment samples), enrichment cultures and subsequent transfers (Transfer 1 and Transfer 2) were analysed by 16S rRNA gene amplicon sequencing (Illumina MiSeq) following the protocol by [26]. The primers 341f (5′-CCT ACG GGN GGC WGC AG-3′) and 785r (5′-GAC TAC HVG GGT ATC TAA KCC-3′) were used to amplify the V3–V4 region of the 16S rRNA genes [27]. The PhiX Control v3 Library was included as a control in accordance with Illumina’s protocol.

Demultiplexed paired-end reads were processed using the DADA2 software package (ver. 1.28.0) [28] and cutadapt (ver. 5.1) [29] to remove primers within the R environment [30]. The following parameters were used to filter and trim the reads: truncLen = c(230,210), maxN = 0, maxEE = c(5,7), and truncQ = 2. Paired-end reads were merged based on sample inference and the learned error rates. The chimaeras were removed using the consensus method. The taxonomic assignment was done using the Silva database (ver 138.1) [31]. The sequences were rarefied to the lowest read numbers of the samples. The read numbers of the inoculum were lower than those of the samples from enrichments and subsequent transfers. Therefore, initially, all samples were rarefied together to enable comparison (rarefied to 3270). Then the samples were analysed into two groups with separate rarefaction: inoculum samples (rarefied to 3270) and the combined enrichment and transfer samples (rarefied to 14,300).

Diversity indices and evenness were calculated using the R package phyloseq (ver 1.50.0) [32]. Beta-diversity was calculated using the Bray–Curtis dissimilarity index based on rarefied genus abundances and visualised as nonmetric multidimensional scaling (NMDS) plots. The sequencing data are deposited in the EMBL-EBI database with the project number PRJEB101631.

#### 2.3.2. Taxonomic Identification of the Selected Isolates

The taxonomic identification of the selected isolates was carried out by partial Sanger sequencing of the 16S rRNA gene. Individual colonies were picked and suspended in 50 µL sterile water and frozen overnight. Following incubation at 95 °C for 3 min, the samples underwent a second freezing step for 20 min. The samples were then thawed and centrifuged at 13,000 rpm. The isolated DNA from the supernatant was diluted 1:10 before being used as a template in PCR.

The 16S rRNA genes were amplified using the universal primers UniBac27f and Univ1492r. PCR was conducted under the following conditions: initial denaturation at 95 °C for 60 s; 30 cycles of denaturation at 95 °C for 15 s, annealing at 58 °C for 10 s, and extension at 72 °C for 15 min; final extension at 72 °C for 15 min. PCR products were purified using the EXSTM Pure Enzymatic PCR Clean-up Kit (NimaGen, Nijmegen, The Netherlands). Briefly, 5 µL PCR product was mixed with 2 µL EXS Pure and incubated at 37 °C for 4 min and then at 90 °C for 1 min. The samples were stored at 4 °C until sequencing. The sequencing was conducted using BigDye Terminator v3.1 Cycle Sequencing Kit (Applied Biosystems following the manufacturer’s protocol) and an ABI PRISM 3130xl Genetic Analyzer (Applied Biosystems, Darmstadt, Germany).

## 3. Results

### 3.1. HOB Enrichment Cultures

**Figure 1:**
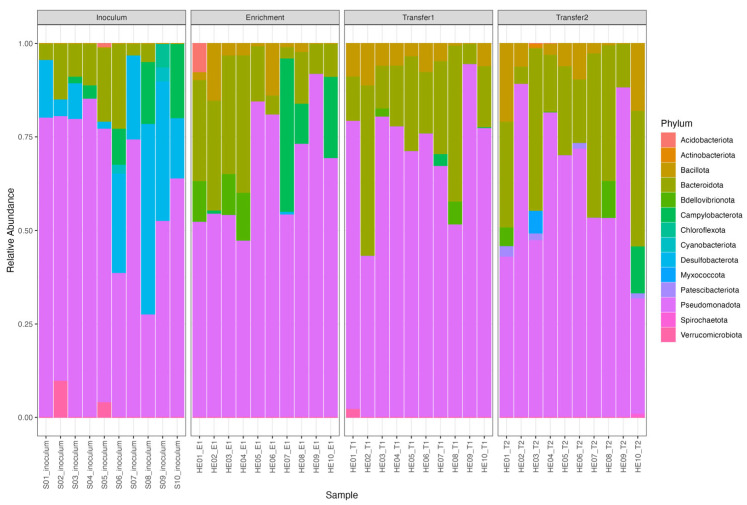
Bacterial community succession during the enrichment of HOB at the phylum level.

The microbial community succession during the HOB enrichment process was assessed by the 16S rRNA gene amplicon sequencing, enabling a direct comparison between inocula, initial enrichments, and subsequent transfers (Figure 1). In general, the inocula were represented by eight phyla, with Pseudomonadota being the predominant group (27–85%; average: 65%), except in samples S06 (38%) and S08 (27%). Desulfobacterota and Bacteroidota were present in most inocula above 4% (except S04, S09, and S10). In contrast, Desulfobacterota composed approximately half of the community of S08, and it was also notably abundant in S09, representing 37% of all sequences. On the other hand, Bacteroidota abundance was the highest in S06 (23%), followed by S05 (20%). S02 was the only inoculum with a notably high abundance of Verrucomicrobiota (10%). Campylobacterota was abundant in S10 (20%), S08 (17%), and S06 (10%). The results suggested a strong source-dependent heterogeneity.

**Figure 2:**
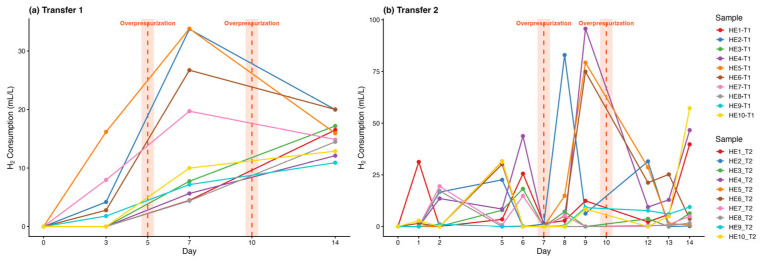
H_2_ consumption (mL/L) of enrichment cultures across successive transfers. (**a**) Transfer 1: measurements taken on Days 0, 3, 7, and 14. (**b**) Transfer 2: measurements taken on Days 0–14. Dashed lines indicate over pressurisations.

Following the first enrichment step, a pronounced shift in community compositions was observed across all samples, indicating a strong selective pressure imposed by H_2_/O_2_/CO_2_ conditions (Figure 2). The shift was mainly driven by changes in the relative abundance of Pseudomonadota and Bacteroidota. As enrichment progressed through subsequent transfers, communities adapted to the selective environment with increased H_2_/O_2_/CO_2_ concentrations, and the communities became progressively simplified with a clear decrease in the number of detected phyla. Notably, Campylobacterota, Chloroflexota, and Cyanobacteriota species diminished after enrichment and did not persist in the subsequent transfers.

The stepwise transition from diverse inoculum to more specialised communities highlights the strong selective effect of the applied cultivation strategy, suggesting that only part of the initial microbial diversity is adapted to hydrogen-oxidising conditions.

**Figure A2:**
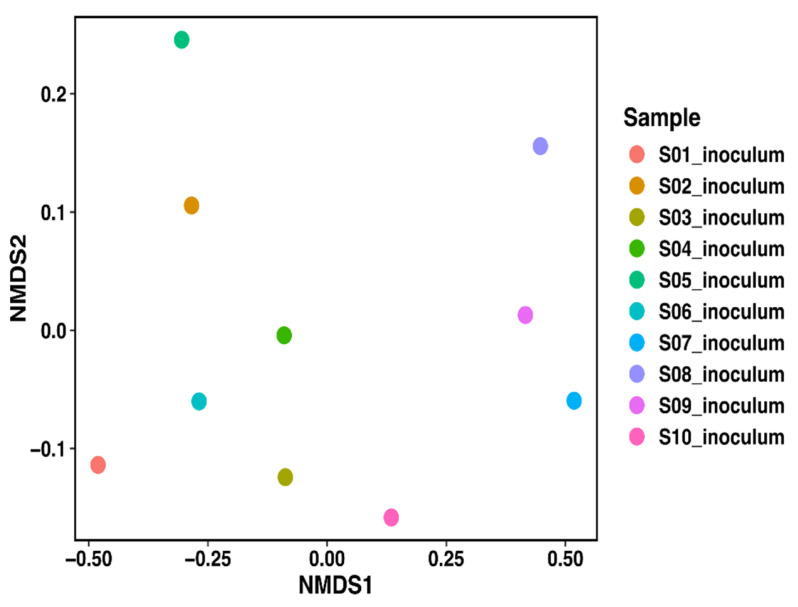
Nonmetric multidimensional scaling (NMDS) plot based on the Bray–Curtis distance showing the bacterial community patterns of the inocula.

The beta-diversity analysis further highlights the pronounced separation among inoculum communities (Appendix A, Figure A2). Nonmetric multidimensional scaling (NMDS) ordination shows a clear spatial variation in samples with no clustering pattern. This indicates a high degree of compositional dissimilarity among the inoculum, reflecting distinct ecological origins. Such variation in the initial microbial communities is important; communities with distinct compositions are likely to yield different enriched communities following selective enrichment processes.

**Figure 3:**
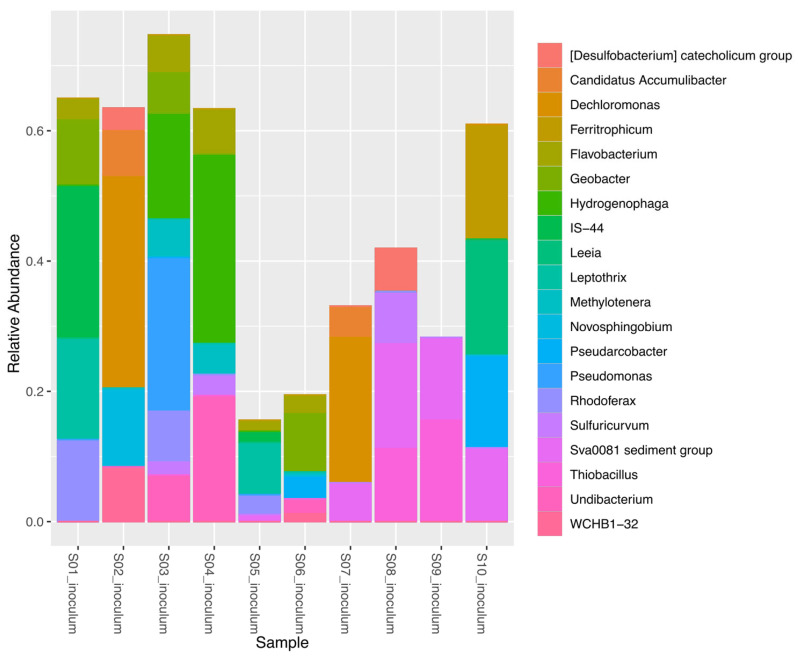
The bacterial communities of the inocula (the top 20 genera).

At the genus level, the inocula showed a distinct community structure linked to specific ecological functions (Figure 3). For instance, the *Thiobacillus* and *Sva0081* sediment group were predominant genera in S08 and S09 (samples taken from the same oligotrophic lake), together representing 62% and 84% of the communities, respectively. On the other hand, *Dechloromonas* was uniquely abundant in S02 (45%) and S07 (50%) (eutrophic pond). *Hydrogenophaga* (S03:19% and S04:36%), while *Leeia* (S10 (oligotrophic lake): 25%) were unevenly distributed across samples. On the other hand, *Leptothrix* comprised approximately 20% of the communities in S01 and S05. Overall, these results revealed that the bacterial communities of the inoculum were highly heterogeneous, and the environmental conditions had strong effects on shaping the community composition.

**Figure 4:**
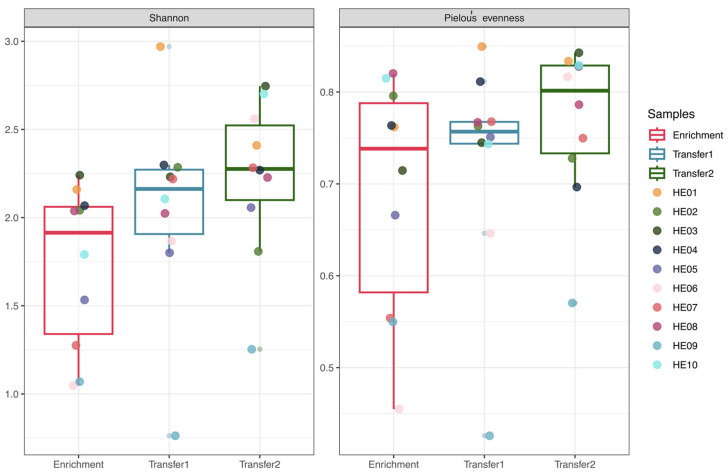
Variation in the bacterial diversity and evenness throughout the enrichment of HOB.

The alpha diversity patterns of the samples revealed heterogeneous responses to the enrichment process (Figure 4). While enrichment is typically expected to reduce the diversity, the observed pattern was sample-specific. Some cultures (such as HE01, HE06, and HE10) showed an increase in diversity after the initial enrichment, followed by stabilisation or a slight decline in the subsequent transfers. Contrarily, the cultures with initially low diversity, such as HE06 and HE09, partially recovered over time, which suggested certain taxa gained an advantage under culture conditions. Although some cultures retained relatively higher diversity, others showed a decrease, which reflects a variational adaptation to culture conditions. The evenness of the communities showed a similar trend to that of the diversity. In some cultures, such as HE02, the diversity and evenness decreased after the second transfer, suggesting successive transfers under selective conditions favoured a smaller number of dominant genera.

**Figure 5:**
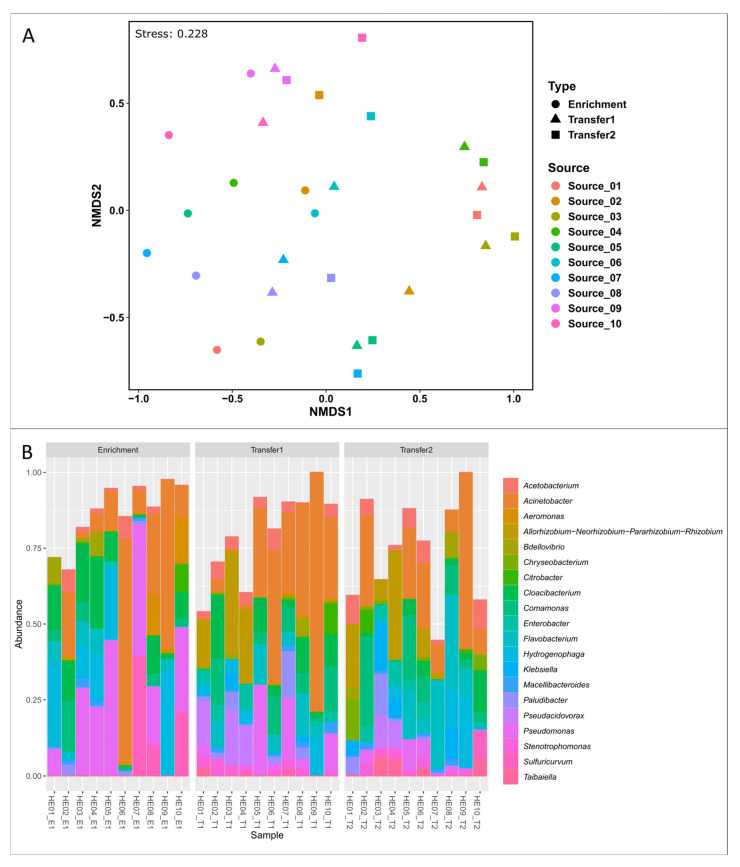
(**A**) Nonmetric multidimensional scaling (NMDS) plot based on the Bray–Curtis distance showing the bacterial community patterns of the first enrichment and subsequent transfers, (**B**) The bacterial communities of the first enrichment and subsequent transfers (the top 20 genera).

The origin of the source had a great influence on bacterial communities even after transfer (Figure 5A). The samples were mostly grouped according to their original source, highlighting the key role of initial community composition. For the cultures from source 9, enrichment, transfer1 and transfer2 were relatively close and showed high stability. On the other hand, for the cultures such as source 1, source 3, source 4, and source 5, the community shifted after the first transfer and did not show significant differences between the transfers.

During enrichment and subsequent transfers, the initial differences resulted in distinct selection. Some genera that were abundant in the inoculum did not persist under enrichment conditions, while others emerged or became dominant only after transfer (Figure 5B). The community showed a clear sample-specific and transfer-specific shift during the enrichment process. *Acinetobacter* species were abundant in the initial enrichment of some cultures, such as HE06 and HE09, then during the subsequent transfer, their relative abundance decreased, and the community shifted towards a more diverse structure. *Acetobacterium* was observed in most of the cultures. *Comamonas* was notably enriched in HE02, HE05 and HE08. On the other hand, *Pseudomonas* species were not enriched under the applied cultivation conditions. Additionally, some genera (such as *Allorhizobium-Neorhizobium-Pararhizobium-Rhizobium*, and *Pseudacidovorax*) showed transient peaks in the first transfer, then decreased or stabilised in the second transfer. This result may be explained by the opportunistic growth followed by the competition and selection of other, more specialist taxa. In the enrichment step, *Cloacibacterium* and *Flavobacterium* were detected in many samples. However, in the later transfers, the members of these genera either disappeared, declined or fell below the detection limit in HE01 and HE03. A similar pattern was observed for *Sulfuricurvum*, which was abundant in the enrichment step of H07, H08, and H10, and the members diminished in the subsequent transfers.

### 3.2. HOB Isolation

Following the second transfer, HOB were successfully recovered from the liquid medium by a single colony isolation technique using solid mineral medium agar and a HOB-supporting gas phase in a specific incubation jar and a total of 70 colonies were selected. Taxonomic profiling based on partial 16S rRNA gene analysis affiliated the strains into 4 genera, namely *Acinetobacter* (50 strains), *Pseudomonas* (18 strains), *Ideonella* (1 strain), and *Klebsiella* (1 strain) (Table 2).

## 4. Discussion

The present study demonstrates that HOB enrichment does not lead to a uniform reduction in microbial diversity but rather drives a restructuring of the community composition in a strong sample-specific pattern.

Consistent with previous reports, HOB are highly diverse, belonging to various phyla including Pseudomonadota (formerly Proteobacteria), Bacillota (formerly Firmicutes), and Actinobacteria [10,20]. In line with this, the enrichment cultures and subsequent transfers in our study were mostly composed of Pseudomonadota, Bacteroidota, and Bacillota members, suggesting that the applied conditions were effectively selected for known and potentially novel hydrogen-oxidising bacteria [10]. On the other hand, progressive simplification of the communities across transfers indicates that only a subset of the initial diversity is competitively adapted to sustained H_2_-based growth conditions.

A key finding of this study is the emergence and persistence of taxa not typically considered as HOB. For instance, initially minor taxa such as *Klebsiella* became abundant in the subsequent transfers, despite limited or no prior evidence of hydrogen-oxidising capacity. Although *Klebsiella* has hydrogenases that are involved in H_2_ production, it is not traditionally classified as HOB [33,34]. Similarly, the presence of *Taibaiella* suggested that current knowledge of HOB diversity may be incomplete and biassed towards well-characterised model organisms like *Cupriavidus necator* [23]. Contrarily, established HOB genera *Hydrogenophaga* [35] proliferated during enrichment under applied conditions, confirming the effectiveness of the cultivation strategy. However, several genera previously reported as representatives of HOB, such as *Pseudomonas*, *Prothomonas*, *Leminobacter*, *Rarobacter*, *Geobacillus*, *Flavobacterium*, *Paracoccus*, *Bacillus*, *Aeromonas*, *Micrococcus*, *Variovorax*, *Mycobacterium*, *Alcaligenes*, *Nocardia*, and *Burkhorderia* [19] were either absent or present at low relative abundances. Only *Pseudomonas*, *Aeromonas* and *Flavobacterium* were found in the top 20 genera. This can be explained by the reflection of the strong culture conditions, which can favour specific functional groups while excluding others, as well as the potential enrichment of novel HOB populations.

The enrichment process also revealed the presence of non-HOB and predatory taxa such as *Bdellovibrio* [36], suggesting that microbial interactions may play a role in shaping community dynamics. Predation-competition and cross feeding could contribute to the restructuring of the community. It should be noted that such potential synergistic interactions within the consortium may have contributed to the growth, where certain strains could have supported the proliferation of the others. Some heterotrophic bacteria may feed on the dead biomass of the primary producer HOB.

Beyond predatory interactions, the transient detection of taxa capable of anaerobic hydrogen metabolism further highlights the complexity of the enrichment process. Hydrogen is a major intermediate in the anaerobic degradation of organic matter. It is used by sulphate-reducing and homoacetogenic bacteria and methanogenic archaea, which can grow chemolithotrophically with H_2_ as their sole energy source [37]. *Acetobacterium*, a homoacetogen, and *Sulfuricurvum*, a facultatively sulphur-oxidising chemolithotroph, were both detected in the early steps of the enrichment process of several cultures and diminished across subsequent transfers. This can be a result of localised anoxic niches within the starting inocula, which may have initially supported anaerobic hydrogen-dependent growth before fully aerobic HO-selective conditions became established. These findings highlighted that hydrogen serves as an electron donor across a broader phylogenetic and metabolic spectrum, and community dynamics in enrichment cultures should also be interpreted in this broader context.

In a previous study, Ehsani and co-workers applied a complicated isolation approach using a heterotroph isolation procedure with plating method (diluted nutrient broth Gelzan™ CM as solidifying agent) and obtained 960 isolates transferred into 96-well microtiter plates, and after several transfers, the hydrogen-oxidising capacity was tested under hydrogen and oxygen stream [35]. Our approach was simpler, utilising the plating method on mineral medium in a custom-made incubation jar with the provided HOB atmosphere.

Several strains closely affiliated with *Acinetobacter* strains were obtained as the most frequent isolates. *Acinetobacter* is a strictly aerobic bacterium that is found in a wide variety of ecosystems, including soils, freshwater, sediments, etc. They are one of the key components in the nutrient cycles thanks to their ability to degrade various organic compounds. Because of their diverse physiological properties, *Acinetobacter* is considered a model organism in environmental sciences, particularly for aromatic compound degradation and natural transformation [38]. From an applied perspective, the isolation of strains such as *Acinetobacter calcoaceticus* raises important biosafety considerations. *Acinetobacter* strains are not considered typical HOB, but an older study [39] described the hydrogen-oxidising ability of certain *Acinetobacter* spp. In this study, three strains were identified as *Acinetobacter calcoaceticus* with 100% similarity (Table 2). *Acinetobacter calcoaceticus* is a member of the *Acinetobacter calcoaceticus-Acinetobacter baumannii* complex, considered an opportunistic pathogen with antibiotic resistance [40]. It is classified as biosafety level 2 [41]; the utilisation of those strains for microbial protein production should not be considered. On the other hand, we isolated 18 strains of *Pseudomonas*, though only a limited number of *Pseudomonas* species was reported as HOB [19], and *Pseudomonas putida* is not among them. In addition, many HOB strains previously affiliated to the genus *Pseudomonas* have been reclassified to the genera *Hydrogenophaga* [42] and *Pelomonas* [43].

*Ideonella* is best known for its specific species, *Ideonella sakaiensis*, which can degrade polyethene terephthalate. A considerable literature has grown up around the theme of PET biodegradation involving this specific species [44,45]. However, two particular strains were characterised as HOB [46,47]. In our study, only one strain was identified as *Ideonella*.

While the isolation of strains affiliated with *Acinetobacter*, *Pseudomonas*, and *Klebsialla* on mineral medium agar under an H_2_/O_2_/CO_2_ conditions is suggestive of hydrogen-oxidising capacity, definitive confirmation of autotrophic HOB metabolism in these lineages would require additional evidence, including confirmed growth in liquid medium in the presence and absence of H_2_, detection of hydrogenase genes and direct quantification of CO_2_ fixation.

The results highlighted that this particular enrichment and isolation strategy, which used mineral medium, a modified anaerobic jar and a gasbag filled with a specific gas mixture, yielded many isolates not previously considered as HOB. On the other hand, Ref. [48] applied a multi-step enrichment method, which includes first the chemolithotrophic medium under a defined gas mixture, followed by plating on both trypticase soy agar and autotrophic media for heterotrophic and autotrophic growth, respectively, and subsequent purification in liquid medium. Overall, this approach is longer and more complex compared to the method used in this study.

## 5. Conclusions

The results highlight two key insights: first the origin of the inoculum plays a decisive role in shaping enrichment results and second, a simple enrichment strategy can uncover previously unrecognised diversity in HOB communities. The enriched cultures and further isolated strains demonstrated hydrogen utilisation, as reflected by forming colonies on mineral medium agar plates when the only carbon source was CO_2_ in the atmosphere (except carbon from agar-agar). Taxonomic analysis revealed that many of these isolates are close relatives of hydrogen-oxidisers frequently reported in the literature, and several of which are known pathogens or opportunistic pathogens. This outlines the significance of experimental validation, as our results confirmed hydrogen metabolism in lineages that are typically not assessed in this context. However, our strategy to obtain colonies on the agar plates was rather selective, since the genus *Acinetobacter* was overrepresented, and HOB genera detected in the enrichment cultures (*Hydrogenophaga*, *Flavobacterium*, *Aeromonas*) were missed by the applied plate streak method. To obtain those specific taxa in pure culture would require further refining of the cultivation (e.g., gas and medium composition, alternative solidifying agent) or screening of a larger number of isolates. Although our results establish a solid basis for future studies, further research work is required on selecting promising strains for in-depth characterisation, particularly on amino acid profiles and metabolic versatility.

## Figures and Tables

**Table 1:** Environmental samples collected for HOB enrichment.

Code	Source	Coordinates	Type of the Source	Type of the Sample	Sampling Date
S01	Kurtköy Stream (Türkiye)	30°11′56″ E, 40°42′19″ N	Stream	Sediment	17 September 2024
S02	Balikhane Stream (Türkiye)	30°8′32″ E, 40°43′4″ N	Stream	Sediment	17 September 2024
S03	Yanikdere Stream (Türkiye)	30°10′30″ E, 40°42′44″ N	Stream	Sediment	17 September 2024
S04	Yanikdere Stream (Türkiye)	30°10′30″ E, 40°42′44″ N	Stream	Sediment	17 September 2024
S05	Waldsee Lauer (Germany)	12°21′22.7″ E, 51°17′07.2″ N	Mesotrophic Lake	Sediment	19 September 2024
S06	Waldsee Lauer (Germany)	12°21′22.7″ E, 51°17′07.2″ N	Mesotrophic Lake	Algae Mat	19 September 2024
S07	Froschteich (Germany)	12°20′42.0″ E, 51°21′33.8″ N	Eutrophic Pond	Sediment	22 September 2024
S08	Kulkwitzer Lake (Germany)	12°15′03.2″ E, 51°18′18.4″ N	Oligotrophic Lake	Rhizosphere	19 September 2024
S09	Kulkwitzer Lake (Germany)	12°15′03.2″ E, 51°18′18.4″ N	Oligotrophic Lake	Sediment	19 September 2024
S010	Haselberg lake (Germany)	12°39′17.7″ E, 51°17′41.2″ N	Mesotrophic Lake	Deep Sediment	21 September 2024

**Table 2:** Taxonomic identification of the isolated pure cultures.

Isolate	Original Source	Closest Cultivable Relative	Sequence Identity	Accession No
I_1	Balikhane Stream (Sakarya, Türkiye), Sediment	*Acinetobacter calcoaceticus*	1	FJ816055.1
I_2	Balikhane Stream (Sakarya, Türkiye), Sediment	*Acinetobacter calcoaceticus*	1	FJ816055.1
I_3	Balikhane Stream (Sakarya, Türkiye), Sediment	*Acinetobacter calcoaceticus*	0.9935	FJ816078.1
I_4	Balikhane Stream (Sakarya, Türkiye), Sediment	*Acinetobacter pittii*	0.9957	FJ860875.1
I_5	Balikhane Stream (Sakarya, Türkiye), Sediment	*Acinetobacter* sp. *PUCM1018*	0.9705	FJ816065.1
I_6	Balikhane Stream (Sakarya, Türkiye), Sediment	*Acinetobacter calcoaceticus*	0.9631	JF683597.1
I_7	Balikhane Stream (Sakarya, Türkiye), Sediment	*Acinetobacter pittii*	0.9851	FJ860875.1
I_8	Balikhane Stream (Sakarya, Türkiye), Sediment	*Acinetobacter calcoaceticus*	0.9979	FJ816055.1
I_9	Balikhane Stream (Sakarya, Türkiye), Sediment	*Acinetobacter calcoaceticus*	1	FJ816055.1
I_10	Balikhane Stream (Sakarya, Türkiye), Sediment	*Acinetobacter pittii*	0.8935	CP033540.1
I_11	Balikhane Stream (Sakarya, Türkiye), Sediment	*Acinetobacter calcoaceticus*	1	FJ816055.1
I_12	Waldsee Lauer (Markkleeberg, Germany) Sediment	*Acinetobacter lwoffii*	0.9723	CP077336.1
I_13	Waldsee Lauer (Markkleeberg, Germany) Sediment	*Acinetobacter* sp. *WCHAc060042*	0.9223	MH428811.1
I_14	Waldsee Lauer (Markkleeberg, Germany) Sediment	*Acinetobacter johnsonii*	0.9726	AM184271.1
I_15	Waldsee Lauer (Markkleeberg, Germany) Sediment	*Acinetobacter johnsonii*	0.9679	AM184271.1
I_16	Waldsee Lauer (Markkleeberg, Germany) Sediment	*Acinetobacter* sp. *WCHAc060042*	0.9868	MH428811.1
I_17	Waldsee Lauer (Markkleeberg, Germany) Sediment	*Acinetobacter* sp. *WCHAc060042*	0.938	MH428811.1
I_18	Waldsee Lauer (Markkleeberg, Germany) Sediment	*Acinetobacter johnsonii*	0.9758	AM184271.1
I_19	Waldsee Lauer (Markkleeberg, Germany) Sediment	*Acinetobacter lwoffii*	0.974	CP077336.1
I_20	Waldsee Lauer (Markkleeberg, Germany) Sediment	*Acinetobacter* sp. *WCHAc060042*	0.9613	MH428811.1
I_21	Waldsee Lauer (Markkleeberg, Germany) Sediment	*Pseudomonas plecoglossicida*	0.9958	KM374717.1
I_22	Waldsee Lauer (Markkleeberg, Germany) Algae mat	*Acinetobacter calcoaceticus*	0.9333	CP070518.1
I_23	Waldsee Lauer (Markkleeberg, Germany) Algae mat	*Acinetobacter* sp. *Z1*	0.9344	CP101115.1
I_24	Waldsee Lauer (Markkleeberg, Germany) Algae mat	*Acinetobacter junii*	0.9851	AM184300.1
I_25	Waldsee Lauer (Markkleeberg, Germany) Algae mat	*Acinetobacter junii*	0.9641	AB859669.1
I_26	Waldsee Lauer (Markkleeberg, Germany) Algae mat	*Acinetobacter lwoffii*	0.9744	CP077336.1
I_27	Waldsee Lauer (Markkleeberg, Germany) Algae mat	*Acinetobacter baumannii*	0.9894	JF919845.1
I_28	Waldsee Lauer (Markkleeberg, Germany) Algae mat	*Acinetobacter sedimenti*	0.9704	MW282900.1
I_29	Waldsee Lauer (Markkleeberg, Germany) Algae mat	*Acinetobacter pittii*	0.9957	CP033525.1
I_30	Waldsee Lauer (Markkleeberg, Germany) Algae mat	*Acinetobacter tandoii*	0.9915	JF304543.1
I_31	Waldsee Lauer (Markkleeberg, Germany) Algae mat	*Acinetobacter calcoaceticus*	1	EU921463.1
I_32	Waldsee Lauer (Markkleeberg, Germany) Algae mat	*Acinetobacter lwoffii*	0.9705	CP077336.1
I_33	Waldsee Lauer (Markkleeberg, Germany) Algae mat	*Acinetobacter junii*	0.981	AM184300.1
I_34	Waldsee Lauer (Markkleeberg, Germany) Algae mat	*Acinetobacter pittii*	0.9979	CP033525.1
I_35	Waldsee Lauer (Markkleeberg, Germany) Algae mat	*Acinetobacter calcoaceticus*	0.9958	EU921463.1
I_36	Froschteich (Leipzig, Germany), Sediment	*Acinetobacter guillouiae*	0.9851	PP813653.1
I_37	Froschteich (Leipzig, Germany), Sediment	*Acinetobacter guillouiae*	0.9957	PP813657.1
I_38	Froschteich (Leipzig, Germany), Sediment	*Pseudomonas delhiensis*	0.9979	FN433047.1
I_39	Froschteich (Leipzig, Germany), Sediment	*Pseudomonas plecoglossicida*	0.9978	CP050291.1
I_40	Froschteich (Leipzig, Germany), Sediment	*Pseudomonas defluvii*	0.9957	CP123067.1
I_41	Froschteich (Leipzig, Germany), Sediment	*Pseudomonas defluvii*	0.9957	CP123067.1
I_42	Froschteich (Leipzig, Germany), Sediment	*Acinetobacter calcoaceticus*	0.9957	KJ767372.1
I_43	Froschteich (Leipzig, Germany), Sediment	*Acinetobacter* sp. *T3-1*	0.9268	KJ127189.1
I_44	Froschteich (Leipzig, Germany), Sediment	*Pseudomonas* sp.	0.9336	PP439520.1
I_45	Froschteich (Leipzig, Germany), Sediment	*Pseudomonas plecoglossicida*	0.9978	OM971641.1
I_46	Froschteich (Leipzig, Germany), Sediment	*Ideonella dechloratans*	0.9711	CP088081.1
I_47	Froschteich (Leipzig, Germany), Sediment	*Pseudomonas plecoglossicida*	0.9979	CP050291.1
I_48	Froschteich (Leipzig, Germany), Sediment	*Pseudomonas plecoglossicida*	0.9895	CP050291.1
I_49	Froschteich (Leipzig, Germany), Sediment	*Pseudomonas plecoglossicida*	0.9957	CP050291.1
I_50	Kulkwitzer Lake (Leipzig, Germany), Rhizosphere	*Acinetobacter calcoaceticus*	0.9872	AB859067.1
I_51	Kulkwitzer Lake (Leipzig, Germany), Rhizosphere	*Pseudomonas migulae*	0.9936	AM114526.1
I_52	Kulkwitzer Lake (Leipzig, Germany), Rhizosphere	*Acinetobacter calcoaceticus*	0.9958	HE801235.1
I_53	Kulkwitzer Lake (Leipzig, Germany), Rhizosphere	*Acinetobacter calcoaceticus*	0.9957	KJ767372.1
I_54	Kulkwitzer Lake (Leipzig, Germany), Rhizosphere	*Acinetobacter calcoaceticus*	0.9979	DQ122365.1
I_55	Kulkwitzer Lake (Leipzig, Germany), Rhizosphere	*Acinetobacter calcoaceticus*	0.9979	HE801235.1
I_56	Kulkwitzer Lake (Leipzig, Germany), Rhizosphere	*Acinetobacter calcoaceticus*	0.9958	HE801235.1
I_57	Kulkwitzer Lake (Leipzig, Germany), Rhizosphere	*Acinetobacter calcoaceticus*	0.9895	HE801235.1
I_58	Kulkwitzer Lake (Leipzig, Germany), Rhizosphere	*Acinetobacter calcoaceticus*	0.9978	KJ767372.1
I_59	Haselberg Lake (Ammelshain, Germany), Deep sediment	*Pseudomonas putida*	0.9957	CP140704.1
I_60	Haselberg Lake (Ammelshain, Germany), Deep sediment	*Klebsiella michiganensis*	0.9936	AP022547.1
I_61	Haselberg Lake (Ammelshain, Germany), Deep sediment	*Acinetobacter pittii*	0.9957	CP033525.1
I_62	Haselberg Lake (Ammelshain, Germany), Deep sediment	*Acinetobacter pittii*	0.9957	CP033525.1
I_63	Haselberg Lake (Ammelshain, Germany), Deep sediment	*Acinetobacter pittii*	0.9979	CP033525.1
I_64	Haselberg Lake (Ammelshain, Germany), Deep sediment	*Pseudomonas putida*	0.9914	CP140704.1
I_65	Haselberg Lake (Ammelshain, Germany), Deep sediment	*Pseudomonas putida*	0.9979	CP140704.1
I_66	Haselberg Lake (Ammelshain, Germany), Deep sediment	*Pseudomonas putida*	0.9893	CP163498.1
I_67	Haselberg Lake (Ammelshain, Germany), Deep sediment	*Pseudomonas kurunegalensis*	0.9957	PQ518700.1
I_68	Haselberg Lake (Ammelshain, Germany), Deep sediment	*Acinetobacter pittii*	0.9915	CP033525.1
I_69	Haselberg Lake (Ammelshain, Germany), Deep sediment	*Pseudomonas batumici*	0.9894	CP144470.1
I_70	Haselberg Lake (Ammelshain, Germany), Deep sediment	*Pseudomonas putida*	0.9915	MZ723875.1

## Data Availability

The sequencing data are deposited in the EMBL-EBI database with the project number PRJEB101631.

## Appendix A

**Table A1:** DSMZ81 mineral medium for chemolithotrophic growth (H-3). Solutions A, B, C are autoclaved separately for 15 min at 121 °C, cooled down to 50 °C and then mixed aseptically with 5.0 mL filter-sterilised standard vitamin solution (see below). The final pH of this medium should be 6.8 without adjustment.

**Solution A:**	**Amount**
KH_2_PO_4_	2.300 g
Na_2_HPO_4_ × 2 H_2_O	2.900 g
Distilled water	50.000 mL
**Solution B:**	**Amount**
NH_4_Cl	1.000 g
MgSO_4_ × 7 H_2_O	0.500 g
CaCl_2_ × 2 H_2_O	0.010 g
MnCl_2_ × 4 H_2_O	0.005 g
NaVO_3_ × H_2_O	0.005 g
Trace element sol. SL-6 (see medium 27)	5.000 mL
Distilled water	920.000 mL
Agar (if necessary)	20.000 g
**Solution C:**	**Amount**
Ferric ammonium citrate	0.050 g
Distilled water	20.000 mL
**Standard vitamin solution:**	**Amount**
Riboflavin	10.000 mg
Thiamine-HCl × 2 H_2_O	50.000 mg
Nicotinic acid	50.000 mg
Pyridoxine-HCl	50.000 mg
Ca-pantothenate	50.000 mg
Biotin	0.100 mg
Folic acid	0.200 mg
Vitamin B12	1.000 mg
Distilled water	100.000 mL
